# The Templating Software Spectrum: Unveiling Variations in the 3D Assessment of Glenoid Morphology in Shoulder Arthroplasty Planning

**DOI:** 10.7759/cureus.102805

**Published:** 2026-02-01

**Authors:** George Matheron, Joshua Ong, Adam Rumian, Angelos Assiotis, Harpal S Uppal

**Affiliations:** 1 Trauma and Orthopaedics, Lister Hospital, Stevenage, GBR

**Keywords:** glenoid morphology, glenoid version, measurement reliability, shoulder arthroplasty/ replacement, three-dimensional preoperative planning, total shoulder arthroplasty

## Abstract

Introduction

Three-dimensional (3D) computed tomography planning improves preoperative quantification of glenoid morphology when planning total shoulder arthroplasty, but measurement reliability across commonly used planning platforms remains uncertain.

Methods

A retrospective within-subject comparison of 30 shoulders was planned on four systems: Stryker Blueprint (BP), Zimmer Signature (ZS), Exactech Equinoxe (EE), and SurgiCase Materialise (MS). Measurements of retroversion and inclination were analysed to evaluate measurement variability and correlations between systems.

Results

Retroversion medians (IQR) were BP −6.5 (10.75), ZS −6.0 (7.0), EE −5.5 (7.0), and MS −3.5 (19.5); overall p=0.37. MS showed greater dispersion and weak correlations with other systems (ρ≈0.03-0.07). Inclination means (SD) were BP 5.93 (8.13), ZS 1.28 (7.27), EE 3.53 (7.57), MS 6.00 (5.07); overall p<0.001. All paired contrasts were significant except for BP vs. MS (−0.07, 95% CI −2.14 to 2.01; p=0.948). Inter-system inclination correlations were strong among BP, EE, and ZS (r≈0.80-0.90) and moderate to strong for MS vs. others (r≈0.57-0.75).

Conclusion

Platforms were not fully interchangeable. Retroversion showed no group-level bias but reduced precision, particularly for MS, while inclination was highly precise yet displayed platform-specific level differences (notably lower with ZS). Based on this, threshold-adjacent decisions (augment selection, baseplate tilt) may vary by platform. Verification of retroversion outliers and confirmation of inclination near boundaries are recommended.

## Introduction

Total shoulder arthroplasty is an established long-term treatment for glenohumeral arthritis and rotator cuff disease, with 9,809 shoulder replacements performed in the United Kingdom in 2024 alone [1-4]. As the population continues to age, utilisation is projected to rise at a faster rate than for hip or knee arthroplasty [5]. In this context, minimising complications, revision surgery, and length of stay is essential to limit the burden upon healthcare systems.

Accurate and reproducible planning is therefore central to this aim [6-8]. In particular, robust characterisation of glenoid orientation - the geometric relationship between the glenoid cavity and the scapular plane - underpins the correction of bone loss, implant/augment selection, and restoration of joint biomechanics. This assessment is frequently complicated by asymmetric wear, osteophytes, and cavitary or segmental bone loss that distort native landmarks and the scapular reference frame [9]. Although debate persists regarding the ideal component positioning (version and inclination), appropriate positioning is essential for balancing forces across the prosthetic joint and ensuring the long-term success of shoulder arthroplasty [10]. Conversely, suboptimal component positioning and excessive glenoid reaming (loss of bone stock) can affect longevity via scapular notching, eccentric loading, instability, glenoid loosening, and eventual failure [7,8,11-13].

Planning has evolved significantly from plain radiography to the introduction of two-dimensional (2D) computed tomography (CT) and, more recently, to three-dimensional (3D) CT-based platforms. While 2D CT improved visualisation of bony anatomy, measurements are sensitive to slice orientation and uncorrected scapular rotation, introducing variability [14,15]. Multiple contemporary 3D planning software programs have been introduced, allowing reorientation of the scapula plane and modelling of bone loss, demonstrating greater accuracy and reproducibility for native glenoid morphology than 2D methods [9,16]. This enhanced anatomical characterisation supports more consistent planning, more appropriate implant selection, and reduced intra-operative variability [17-19]. Alongside improvements in the understanding of arthroplasty biomechanics, the increase in planning accuracy has seen improvements in surgical outcomes, particularly early implant failure [11,12,20,21]. However, despite widespread adoption, limited information exists regarding the measurement variability between commonly used systems, raising practical questions about reliability and interchangeability between platforms [22,23]. Accordingly, this study aims to evaluate and compare four widely available 3D CT-guided templating platforms for their agreement on native glenoid retroversion and inclination measurements. By doing so, we aim to provide surgeons with practical insight into the degree of agreement and interchangeability between these systems for shoulder arthroplasty planning.

## Materials and methods

A retrospective review was conducted of 30 consecutive patients who underwent pre-operative CT in preparation for shoulder arthroplasty. All studies were performed at the same institution using a standardised protocol, with thin-slice imaging of the entire scapula to ensure comprehensive visualisation and consistent scapular-plane referencing.

**Figure 1:**
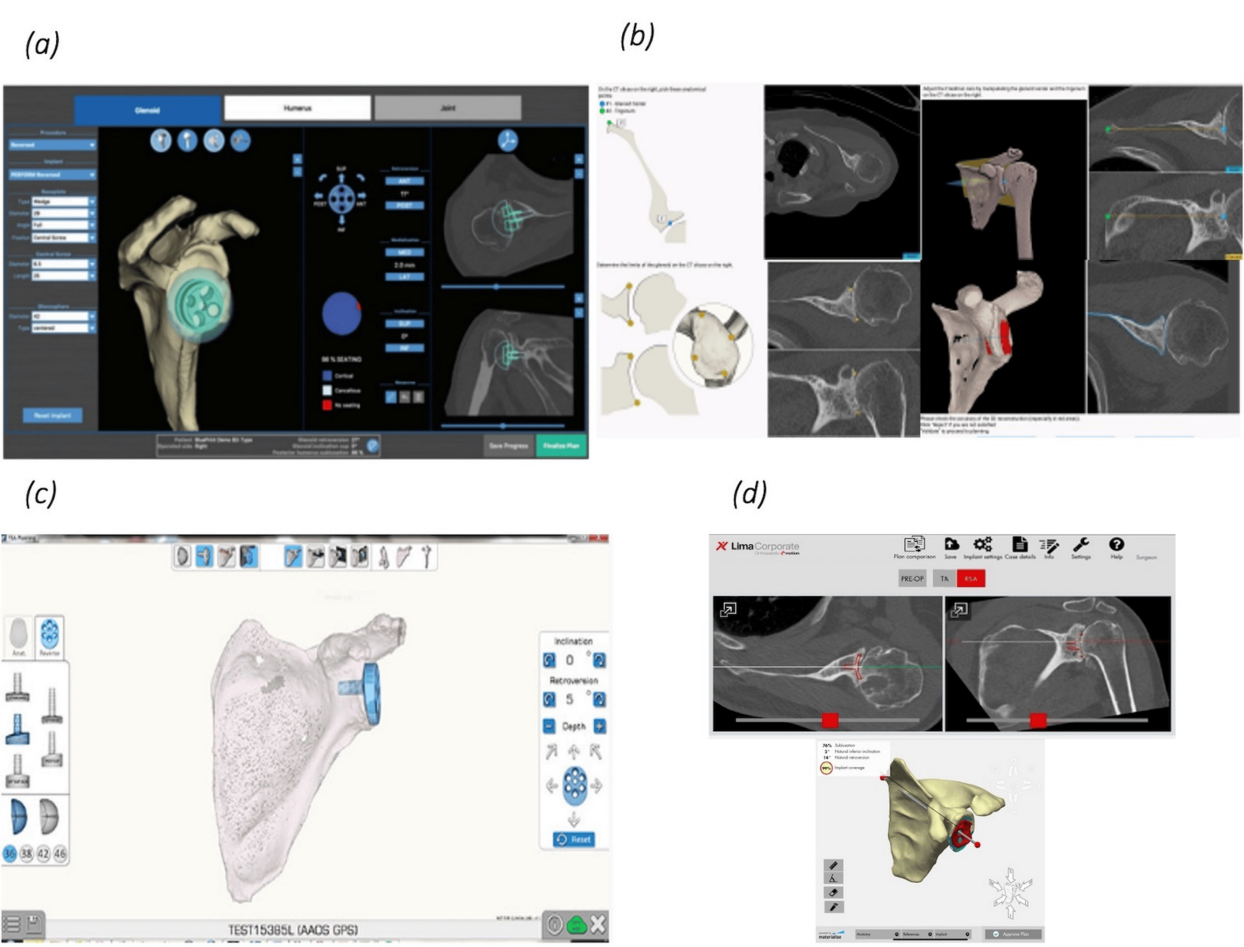
Planning software systems used: (a) Blueprint 3D by Stryker – BP, (b) Zimmer Signature – ZS, (c) Exactech Equinoxe GPS – EE, (d) Materialise SurgiCase – MS

Each shoulder was planned on four commonly used pre-operative planning systems to obtain measurements of native glenoid version and inclination. Three of the systems employ automated, manufacturer-developed preoperative planning solutions: Stryker's Blueprint 3D planning (BP), Zimmer Signature ONE Surgical Planning System (ZS), and Materialise SurgiCase by Lima-Enovis (MS) (Figure 1).

BP and ZS derive scapular planes and native orientation using proprietary automated algorithms that collect thousands of data points on the scapula body, calculating the scapula planes according to those boundary median points. These automated vector calculations of the model are verified by technical engineers. MS generates automated measurements and analysis, with a human post-processing review. The authors do not have access to the underlying algorithm used to determine the scapula plane and glenoid morphology for these systems. The final platform utilised was Exactech Equinoxe GPS (EE), a surgeon-directed system. The surgeon uploads the CT, selects reference points, including the glenoid centre, trigonum, and glenoid limits, and adjusts the Friedman axis (glenoid centre to the medial scapular border). The resulting 3D reconstruction is then confirmed by the surgeon, from which implant selection can then be performed.

Statistical analysis

Distributional assumptions were assessed with the Shapiro-Wilk test. Retroversion deviated from normality, and therefore, the Friedman's test for the omnibus repeated-measures comparison was used, and where applicable, Wilcoxon signed-rank tests for within-subject pairwise contrasts. For inclination, normality was verified with the Shapiro-Wilk test, and sphericity was acceptable on Mauchly’s W test. Therefore, a repeated-measures ANOVA (rANOVA) was used for the omnibus comparison across systems. When the omnibus test was significant, within-subject paired t-tests quantified pairwise differences. Where adjustment for multiplicity was desired, the Holm correction was applied. Agreement between systems for inclination was quantified with Pearson’s correlation (r). Correlation was judged as very strong from 1 to 0.9, strong from 0.9 to 0.7, moderate from 0.7 to 0.5, low from 0.5 to 0.3, and poor from 0.3 to 0. The alpha risk was set to 0.05. All analyses used EasyMedStat (version 3.44; www.easymedstat.com).

## Results

**Figure 2:**
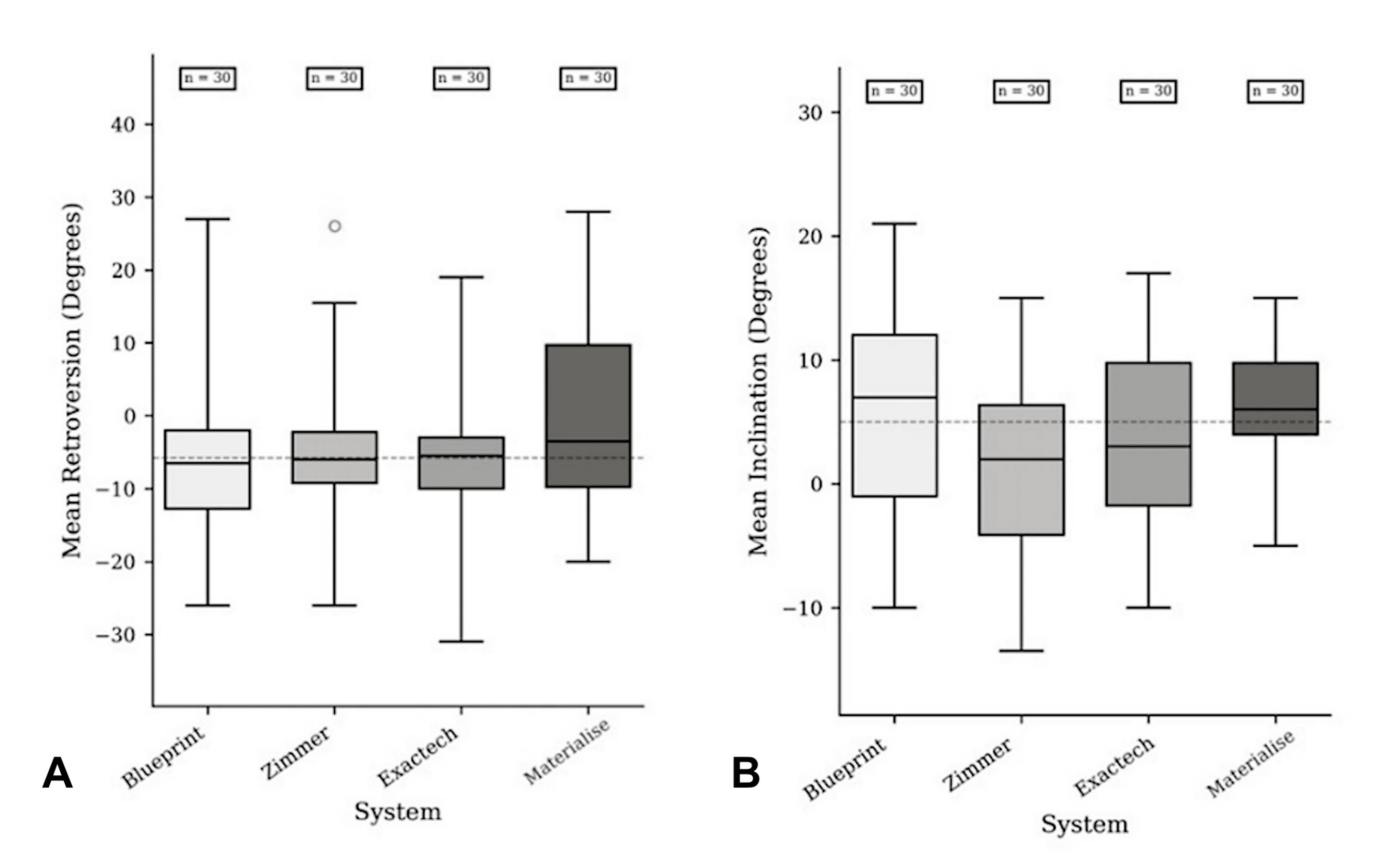
Distribution of (A) retroversion and (B) inclination across systems (box & whisker)

A total of 30 shoulders were analysed (nine male, 21 female), at a mean of 73 (range, 45-88). The right shoulders comprised 10 patients, with the remaining 20 left-sided. Median retroversion (IQR) was −6.5 (10.75) for BP, −6.0 (7.0) for ZS, −5.5 (7.0) for EE, and −3.5 (19.5) for MS (Table 1). The repeated-measures omnibus (Friedman) test showed no overall difference among systems (χ² = 3.14, p = 0.37). Observed median paired differences were small, and in keeping with the non-significant omnibus, inferential post-hoc testing was not interpreted. Distributions are shown in Figure 2, with paired analyses presented in Table 2 for completeness.

**Table 1:** Measurements of retroversion and inclination for the four software systems

	Mean	Median	Interquartile Range (Q1,3)	Standard Deviation (SD)	95% Confidence Interval (CI)
Retroversion					
Blueprint	-6.33	-6.50	-12.75, -2.00	9.64	-9.93, -2.73
Exactec	-5.83	-5.50	-10.00, -3.00	9.15	-9.25, -2.42
Zimmer	-5.10	-6.00	-9.25, -2.25	9.58	-8.68, -1.52
Materialise	-0.73	-3.50	-9.75, 9.75	11.62	-5.07, 3.61
Inclination					
Blueprint	5.93	7	-1.00, 12.00	8.13	2.90, 8.97
Exactec	3.53	3	-1.75, 9.75	7.57	0.71, 6.36
Zimmer	1.28	2	-4.12, 6.38	7.27	-1.43, 4.00
Materialise	6	6	4.90, 9.75	5.07	4.11, 7.89

**Table 2:** Groupwise comparison of retroversion and inclination measurements

	Native Retroversion (Mean Paired Difference, Degrees)	p-value	Native Inclination (Median Paired Difference, Degrees)	p-value
Blueprint vs. Materialise	0.00	0.361	0.07	0.948
Exactec vs. Materialise	-1.50	0.492	2.47	0.039
Zimmer vs. Materialise	-2.50	0.948	4.72	<0.001
Blueprint vs. Zimmer	-0.50	0.068	-4.65	<0.001
Blueprint vs. Exactec	0.00	0.427	2.40	0.009
Zimmer vs. Exactec	0.50	0.186	-2.25	0.013

Mean inclination (SD) measured by BP was 5.93 (8.13), EE 3.53 (7.57), ZS 1.28 (7.27), and MS 6.00 (5.07). Repeated-measures ANOVA demonstrated a significant main effect of system (p<0.001). Pre-specified within-subject post-hoc paired t-testing showed that BP yielded a greater inclination than ZS by 4.65 (95% CI 3.33 to 5.97; p<0.001) and greater than EE by 2.40 (95% CI 0.64 to 4.16; p=0.009) with no difference from MS (−0.07; 95% CI −2.14 to 2.01; p=0.948). ZS produced a lower inclination than EE by 2.25 (95% CI −3.99 to −0.51; p=0.013) and lower than MS by 4.72 (95% CI −6.51 to −2.92; p<0.001). MS exceeded EE by 2.47 (95% CI 0.13 to 4.80; p=0.039). Findings were unchanged when applying a Holm adjustment across the six paired comparisons. These contrasts indicate systematically lower inclination estimates with ZS compared with BP and MS (and lower than EE), with BP and MS similar and EE intermediate. The inclination distribution is shown in Figure 2, and full pairwise results are provided in Table 2.

Correlations

For retroversion (Spearman ρ), inter-system agreement was strong among three platforms: BP vs. ZS (ρ=0.91; r²=0.89), BP vs. EE (ρ=0.84; r²=0.82), and ZS vs. EE (ρ=0.89; r²=0.90); all p<0.001 (Table 3). In contrast, correlations between MS and each of the other platforms were weak (ρ≈0.03-0.07; r²≈0.00-0.01; p>0.70). For inclination (Pearson r), correlations were strong between BP and ZS (r≈0.90; r²≈0.81), BP and EE (r≈0.82; r²≈0.68), and ZS and EE (r≈0.80; r²≈0.64), all p<0.001. Associations involving MS were moderate to strong (MS-BP r≈0.74; r²≈0.54; MS-ZS r≈0.75; r²≈0.57; MS-EE r≈0.57; r²≈0.33; all p≤0.001).

**Table 3:** Groupwise correlation coefficient values for retroversion and inclination

	p-value	r^2^ value	p-value
Retroversion			
Blueprint vs. Materialise	0.026	<0.001	0.893
Exactec vs. Materialise	0.087	0.008	0.646
Zimmer vs. Materialise	0.053	0.001	0.779
Blueprint vs. Zimmer	0.910	0.885	<0.001
Blueprint vs. Exactec	0.840	0.824	<0.001
Zimmer vs. Exactec	0.890	0.904	<0.001
Inclination			
Blueprint vs. Materialise	0.740	0.544	<0.001
Exactec vs. Materialise	0.570	0.329	<0.001
Zimmer vs. Materialise	0.750	0.565	<0.001
Blueprint vs. Zimmer	0.910	0.810	<0.001
Blueprint vs. Exactec	0.820	0.676	<0.001
Zimmer vs. Exactec	0.800	0.644	<0.001

## Discussion

Accurate pre-operative characterisation of glenoid version and inclination underpins the correction of bone loss, implant/augment selection, restoration of biomechanics, and reproducible reporting across centres [12,13]. Malposition, particularly retroversion >10° or abnormal inclination, has been associated with eccentric loading, micromotion, inferior impingement and notching, erosion, and early loosening [12,21,24,25]. Excessive glenoid inclination is likewise linked to inferior impingement and notching, glenoid neck erosions, and prosthesis loosening [7,26,27].

In this within-subject comparison across four planning platforms, retroversion did not differ at the group level, and the paired median differences were small, indicating no systematic inter-platform bias. However, MS demonstrated markedly greater dispersion (IQR 19.5 vs. 7-11 for other platforms) and weak inter-system correlations with the others (ρ≈0.03-0.07). This combination of no average shift but lower precision suggests that MS measurements are not consistently higher or lower relative to other systems, yet for an individual patient, the value may be unusually high or low and may not align with the value another platform would report.

By contrast, inclination showed a clear system effect. Repeated-measures ANOVA identified significant differences (p<0.001), and within-subject tests showed that all pairwise contrasts were significant except BP vs. MS (p=0.948). BP was higher than ZS by 4.65 and EE by 2.40, MS was higher than ZS by 4.72 and than EE by 2.47, and EE exceeded ZS by 2.25. Despite these level differences, inter-system correlations were strong, indicating good precision with non-zero bias such that the systems track patients similarly but with differing absolute values. These findings are consistent with prior work, showing that inter-platform measurements can be highly correlated yet differ in absolute level, particularly for inclination. Sabesan et al. reported a reliable cross-platform assessment of version (p>0.05) with strong correlation (r≈0.7) but a significant difference in inclination between automated and surgeon-directed workflows (p=0.039) despite only moderate correlation (r≈0.6) [23]. Our results mirror this pattern in which inclination comparisons between automated systems were significant (p<0.001), with smaller yet still significant differences when a surgeon-directed workflow was involved (BP vs. EE p=0.009; ZS vs. EE p=0.013), whereas the version showed no group-level difference.

Taken together, these data suggest that while 3D planning outperforms 2D slice measurement for version, inclination, and bone loss, platforms may not be truly interchangeable. They tend to track patients similarly (high precision) yet sit at different absolute levels (bias). Because inclination thresholds often guide augment selection and baseplate tilt, platform-specific bias can move a case across a threshold when decisions hinge on a few degrees. Observed differences likely reflect variations in calculation parameters or landmark selection. Reference points for inclination and retroversion may be distorted by glenoid cavity changes such as osteophytes, erosions, or superior-to-inferior twists of the glenoid [28,29]. Re-orienting CT data into a reproducible scapular plane reduces variability, but divergence between proprietary algorithms and user-directed steps remains [27]. Surgeon-directed systems offer more flexibility but may introduce surgeon error, whereas automated systems rely on their underlying algorithms, making the process of glenoid mapping less transparent. Finally, as implantation typically deviates little from the pre-operative plan, platform-related differences are not only academic. They can translate into different augment choices or baseplate tilt for the same shoulder [16]. Although variation exists between implant design manufacturers, there should be no statistical difference in measurement parameters to allow consistency in the implementation of the biomechanical principles of shoulder replacement. Therefore, it is important to recognise that platform-specific differences exist and should be factored into decision-making. Two practical points follow. First, for retroversion, particularly when an MS value is near a decision threshold or appears incongruent with the CT anatomy, internal verification is appropriate with repetition or refinement of segmentation, confirmation of rim landmarks, and re-checking of scapular plane derivation, then considering the value within a narrow sensitivity range rather than as a rigid cut-off. Secondly, for inclination, known offsets between platforms mean that threshold-adjacent plans should explicitly confirm the reference frame and landmarking within the chosen platform, and cases that are close to a decision threshold should be reviewed carefully.

The strengths of this study include a within-subjects design across four widely used systems (both fully automated and surgeon-directed) and the use of a standardised whole-scapula CT protocol at a single site, minimising inter-patient variation in imaging technique. Limitations include a modest sample size, lack of access to proprietary algorithms, and absence of an external ground truth (e.g., cadaveric validation). Similarly, CT scan variation, artefact, or differences in how reference points used to calculate version and inclination may introduce measurement variability. Moreover, surgeon-directed software may introduce subjectivity and human error, leading to inter-user variability. Additionally, intra-operative execution or clinical outcomes were not assessed, noting prior evidence that implantation typically follows the pre-operative plan closely [16]. Accordingly, our results reflect planning agreement rather than implant placement or patient-reported results, and further research is needed to better understand the downstream effects of such variability.

## Conclusions

Across four contemporary planning systems, retroversion showed no group-level bias but reduced inter-system precision, particularly for MS, whereas inclination was highly precise yet systematically different between pairs. Surgeons should account for these platform-specific behaviours during pre-operative planning by verifying retroversion values that drive correction and confirming inclination decisions close to thresholds so that implant selection and baseplate orientation remain guided by anatomy and informed by known characteristics of the planning platform.
